# Supplementary material for: Next-generation sequencing of BRCA1 and BRCA2 genes for rapid detection of germline mutations in hereditary breast/ovarian cancer
Source: PeerJ. 2019 Apr 22;7:e6661. doi: 10.7717/peerj.6661 (PMC6482939; doi:10.7717/peerj.6661)
Supplement: Supplemental Information 1 — Variants identified by NGS [file peerj-07-6661-s001.docx]

|  | ***BRCA1*** | ***BRCA2*** |
| --- | --- | --- |
| **missense** | c.1067A>G (p.Gln356Arg) c.2077G>A (p.Asp693Asn)  c.2612C>T (p.Pro871Leu) c.3113A>G (p.Glu1038Gly) c.3548A>G (p.Lys1183Arg)  c.4837A>G (p.Ser1613Gly) c.4956G>A (p.Met1652Ile) | c.1114A>C (p.Asn372His)  c.2971A>G (p.Asn991Asp)  c.865A>C (p.Asn289His) |
| **synonymous** | c.2082C>T (p.Ser694=) c.2311T>C (p.Leu771=)  c.4308T>C (p.Ser1436=) | c.1365A>G (p.Ser455=)  c.2229T>C (p.His743=)  c.3396A>G (p.Lys1132=) c.3807T>C (p.Val1269=)  c.4563A>G (p.Leu1521=) c.6513G>C (p.Val2171=)  c.7008C>T (p.Arg2336=) c.7242A>G (p.Ser2414=) |
| **intronic** | c.-19-115T>C c.441+51T>C  c.442-34C>T  c.5075-53C>T | c.1910-51G>T c.1910-74T>C  c.425+67A>C  c.681+56C>T c.6841+78_6841+81delAATT c.6841+80_6841+83delTTAA c.7806-14T>C c.8755-66T>C c.9257-113T>G c.9257-16T>C |
| **untranslated region (5’ UTR)** |  | c.-26G>A |
| **1 nonsense (benign)** |  | c.9976A>T (p.Lys3326Ter) |
