# Supplementary material for: Next-generation sequencing of BRCA1 and BRCA2 genes for rapid detection of germline mutations in hereditary breast/ovarian cancer
Source: PeerJ. 2019 Apr 22;7:e6661. doi: 10.7717/peerj.6661 (PMC6482939; doi:10.7717/peerj.6661)
Supplement: Supplemental Information 2 — Run data of the Training and Validation samples sequenced on Ion 314 Chip. [file peerj-07-6661-s002.docx]

| **Sample ID** | **Mapped Reads** | **On Target** | **Mean depth** | **Uniformity** |
| --- | --- | --- | --- | --- |
| **BR760** | 142412 | 96% | 869 | 96% |
| **BR767** | 150897 | 97% | 858 | 91% |
| **BR771** | 139600 | 95% | 816 | 97% |
| **BR781** | 133859 | 93% | 783 | 98% |
| **BR782** | 123890 | 94% | 739 | 98% |
| **BR784** | 136838 | 93% | 798 | 98% |
| **BR787** | 137175 | 93% | 804 | 99% |
| **BR840** | 77528 | 94% | 462 | 98% |
| **BR847** | 78806 | 93% | 457 | 97% |
| **BR852** | 74848 | 94% | 443 | 99% |
| **BR864** | 70954 | 95% | 419 | 97% |
| **BR867** | 76413 | 94% | 448 | 98% |
| **BR877** | 81407 | 93% | 471 | 97% |
| **BR898** | 112294 | 94% | 646 | 97% |
| **BR908** | 113313 | 95% | 665 | 98% |
| **BR913** | 128511 | 94% | 737 | 97% |
| **BR914** | 148806 | 92% | 803 | 98% |
| **BR915** | 117822 | 92% | 634 | 96% |
| **BR918** | 153125 | 91% | 811 | 96% |
| **BR921** | 77146 | 91% | 421 | 96% |
| **BR926** | 68739 | 92% | 385 | 97% |
| **BR929** | 82494 | 91% | 450 | 97% |
| **BR934** | 73678 | 90% | 399 | 97% |
| **BR937** | 155407 | 99% | 979 | 97% |
| **BR938** | 162775 | 99% | 1026 | 98% |
| **BR944** | 148884 | 98% | 922 | 97% |
| **BR956** | 174878 | 98% | 1070 | 97% |
| **BR962** | 178839 | 98% | 1067 | 97% |
| **BR965** | 172637 | 98% | 1046 | 98% |
| **BR917** | 137737 | 99% | 880 | 97% |
| **BR977** | 159704 | 98% | 1011 | 98% |
| **BR968** | 154071 | 99% | 962 | 97% |
| **BR969** | 166926 | 98% | 1049 | 98% |
| **BR971** | 163252 | 98% | 1002 | 97% |
| **BR972** | 145807 | 98% | 896 | 97% |
| **BR974** | 158902 | 99% | 979 | 97% |
| **BR975** | 157708 | 97% | 952 | 97% |
| **BR979** | 169820 | 98% | 1053 | 97% |
| **BR982** | 139328 | 98% | 865 | 97% |
| **BR987** | 150199 | 98% | 921 | 97% |
| **BR988** | 131492 | 97% | 806 | 97% |
| **BR983** | 104039 | 99% | 654 | 96% |
| **BR990** | 108086 | 98% | 674 | 97% |
| **BR999** | 39816 | 99% | 244 | 97% |
| **BR1000** | 107170 | 99% | 674 | 97% |
| **BR1037** | 87200 | 97% | 535 | 96% |
| **BR1052** | 90825 | 98% | 559 | 97% |
| **BR1058** | 88781 | 98% | 546 | 97% |
| **BR1059** | 92953 | 98% | 577 | 98% |
| **BR1200** | 95229 | 98% | 577 | 98% |
| **BR1224** | 103986 | 98% | 640 | 99% |
| **BR1244** | 109205 | 98% | 632 | 95% |
| **BR1254** | 100748 | 98% | 620 | 99% |
| **BR1278** | 99667 | 97% | 575 | 98% |
| **BR1291** | 111582 | 98% | 666 | 99% |
| **BR1300** | 111287 | 96% | 622 | 95% |
| **BR1301** | 113794 | 95% | 637 | 97% |
| **BR1290** | 124365 | 97% | 730 | 97% |
| **BR1306** | 97883 | 98% | 574 | 98% |
| **BR1307** | 119160 | 99% | 684 | 95% |
| **BR1311** | 118007 | 98% | 723 | 99% |
| **BR58** | 322003 | 94% | 1876 | 96% |
| **BR136** | 259389 | 96% | 1441 | 86% |
| **BR311** | 130799 | 93% | 764 | 98% |
| **BR201** | 132957 | 95% | 798 | 97% |
| **BR407** | 103496 | 94% | 620 | 98% |
| **BR13** | 54784 | 96% | 334 | 98% |
| **BR283** | 86787 | 94% | 487 | 96% |
| **BR281** | 96908 | 93% | 550 | 97% |
| **BR328** | 98614 | 94% | 553 | 97% |
| **BR409** | 93922 | 88% | 509 | 99% |
| **BR59** | 68813 | 94% | 390 | 92% |
| **BR1323** | 90720 | 99% | 547 | 97% |
| **BR1236** | 88228 | 97% | 526 | 97% |
| **BR1271** | 91552 | 98% | 551 | 97% |
| **BR1333** | 92822 | 98% | 567 | 97% |
| **BR1339** | 203839 | 98% | 1274 | 98% |
| **BR1340** | 237281 | 98% | 1501 | 99% |
|  |  |  |  |  |
|  | **Mapped Reads** | **On Target** | **Mean Depth** | **Uniformity** |
| **Average** | 122109 | 96% | 726 | 97% |
| **Min** | 39816 | 88% | 244 | 86% |
| **Max** | 322003 | 99% | 1876 | 99% |
| **SD** | 43431 | 0,03 | 266 | 0,02 |
