# Supplementary material for: Next-generation sequencing of BRCA1 and BRCA2 genes for rapid detection of germline mutations in hereditary breast/ovarian cancer
Source: PeerJ. 2019 Apr 22;7:e6661. doi: 10.7717/peerj.6661 (PMC6482939; doi:10.7717/peerj.6661)
Supplement: Supplemental Information 3 — Run data of the Validation samples sequenced on Ion 316 Chip. [file peerj-07-6661-s003.docx]

| **Sample ID** | **Mapped Reads** | **On Target** | **Mean depth** | **Uniformity** |
| --- | --- | --- | --- | --- |
| **BR1005** | 279770 | 99% | 1719 | 97% |
| **BR1008** | 265299 | 98% | 1655 | 98% |
| **BR1009** | 235961 | 99% | 1462 | 94% |
| **BR1023** | 253562 | 99% | 1590 | 97% |
| **BR1026** | 264723 | 99% | 1660 | 97% |
| **BR1027** | 250625 | 99% | 1587 | 98% |
| **BR1028** | 273322 | 99% | 1714 | 97% |
| **BR1029** | 268342 | 99% | 1674 | 97% |
| **BR1063** | 279218 | 95% | 1722 | 98% |
| **BR1066** | 258157 | 92% | 1498 | 97% |
| **BR1073** | 249163 | 94% | 1514 | 98% |
| **BR1074** | 277834 | 93% | 1639 | 97% |
| **BR1086** | 240733 | 94% | 1412 | 97% |
| **BR1089** | 236905 | 93% | 1406 | 97% |
| **BR1096** | 240460 | 94% | 1411 | 96% |
| **BR1097** | 208899 | 92% | 1222 | 98% |
| **BR1101** | 123327 | 99% | 759 | 97% |
| **BR1106** | 132534 | 98% | 826 | 97% |
| **BR1109** | 111615 | 90% | 642 | 99% |
| **BR1110** | 113482 | 96% | 691 | 98% |
| **BR1111** | 131951 | 99% | 818 | 97% |
| **BR1115** | 116474 | 97% | 717 | 98% |
| **BR1117** | 115519 | 98% | 723 | 97% |
| **BR1125** | 90026 | 98% | 562 | 97% |
| **BR1131** | 231566 | 96% | 1437 | 98% |
| **BR1132** | 247894 | 94% | 1466 | 98% |
| **BR1134** | 220505 | 97% | 1378 | 99% |
| **BR1140** | 310206 | 95% | 1890 | 97% |
| **BR1099** | 290963 | 96% | 1748 | 96% |
| **BR1142** | 292626 | 94% | 1711 | 97% |
| **BR1144** | 216087 | 98% | 1370 | 98% |
| **BR1151** | 276204 | 94% | 1658 | 98% |
| **BR1159** | 276,405 | 96% | 1642 | 98% |
| **BR1167** | 256826 | 95% | 1530 | 98% |
| **BR1168** | 257561 | 96% | 1515 | 98% |
| **BR1177** | 208243 | 95% | 1221 | 99% |
| **BR1184** | 238817 | 98% | 1457 | 98% |
| **BR1187** | 196992 | 95% | 1174 | 98% |
| **BR1141** | 271273 | 97% | 1631 | 98% |
| **BR1194** | 210022 | 98% | 1258 | 97% |
| **BR1201** | 258845 | 96% | 1518 | 97% |
| **BR1203** | 238724 | 98% | 1429 | 98% |
| **BR1204** | 231057 | 97% | 1378 | 98% |
| **BR1206** | 243713 | 98% | 1492 | 98% |
| **BR1209** | 253122 | 98% | 1530 | 98% |
| **BR1213** | 197675 | 97% | 1202 | 98% |
| **BR1208** | 253013 | 98% | 1496 | 99% |
| **BR1212** | 353373 | 99% | 2094 | 96% |
| **BR1223** | 324084 | 95% | 1871 | 96% |
| **BR1215** | 281750 | 98% | 1576 | 92% |
| **BR1233** | 340321 | 99% | 2113 | 98% |
| **BR1234** | 334067 | 99% | 1965 | 92% |
| **BR1235** | 293669 | 98% | 1805 | 98% |
| **BR1237** | 349849 | 99% | 2138 | 95% |
| **BR1182** | 367801 | 97% | 2236 | 98% |
| **BR1238** | 260317 | 98% | 1578 | 97% |
| **BR1248** | 307323 | 98% | 1851 | 96% |
| **BR1261** | 282764 | 98% | 1670 | 96% |
| **BR1267** | 266270 | 99% | 1654 | 98% |
| **BR1268** | 321242 | 99% | 1924 | 97% |
| **BR1270** | 298907 | 97% | 1799 | 98% |
| **BR1272** | 302720 | 98% | 1833 | 97% |
| **BR1283** | 313842 | 98% | 1914 | 98% |
|  |  |  |  |  |
|  | **Mapped Reads** | **On Target** | **Mean Depth** | **Uniformity** |
| **Average** | 244737 | 97% | 1504 | 97% |
| **Min** | 276,405 | 90% | 562 | 92% |
| **Max** | 367801 | 99% | 2236 | 99% |
| **SD** | 70417 | 0,02 | 379 | 0,01 |
