# Supplementary material for: Next-generation sequencing of BRCA1 and BRCA2 genes for rapid detection of germline mutations in hereditary breast/ovarian cancer
Source: PeerJ. 2019 Apr 22;7:e6661. doi: 10.7717/peerj.6661 (PMC6482939; doi:10.7717/peerj.6661)
Supplement: Supplemental Information 4 — 60 BRCA1 and BRCA2 negative samples, with a higher expectation to be carrier of pathogenic mutations [file peerj-07-6661-s004.docx]

|  | ***BRCA1*** | ***BRCA2*** | ***BRCA1*+*BRCA2*** |
| --- | --- | --- | --- |
| Total variants | 404 | 479 | 883 |
| Called positions per patient | 5989 | 10257 | 16246 |
| Total called positions | 359340 | 615420 | 974760 |
| TP | 400 | 466 | 866 |
| FP | 4 | 13 | 17 |
| TN | 358936 | 614941 | 973877 |
| FN | 0 | 0 | 0 |
| Sensitivity, TP/(TP + FN) | 100% | 100% | 100% |
| Specificity, TN/(TN + FP) | 100% | 100% | 100% |
| Positive predictive value (PPV), TP/(TP + FP) | 99% | 97% | 98% |
| Negative predictive value (NPV), TN/(TN + FN) | 100% | 100% | 100% |
