# Supplementary figures and images for: Next-generation sequencing of BRCA1 and BRCA2 genes for rapid detection of germline mutations in hereditary breast/ovarian cancer
Source: PeerJ. 2019 Apr 22;7:e6661. doi: 10.7717/peerj.6661 (PMC6482939; doi:10.7717/peerj.6661)

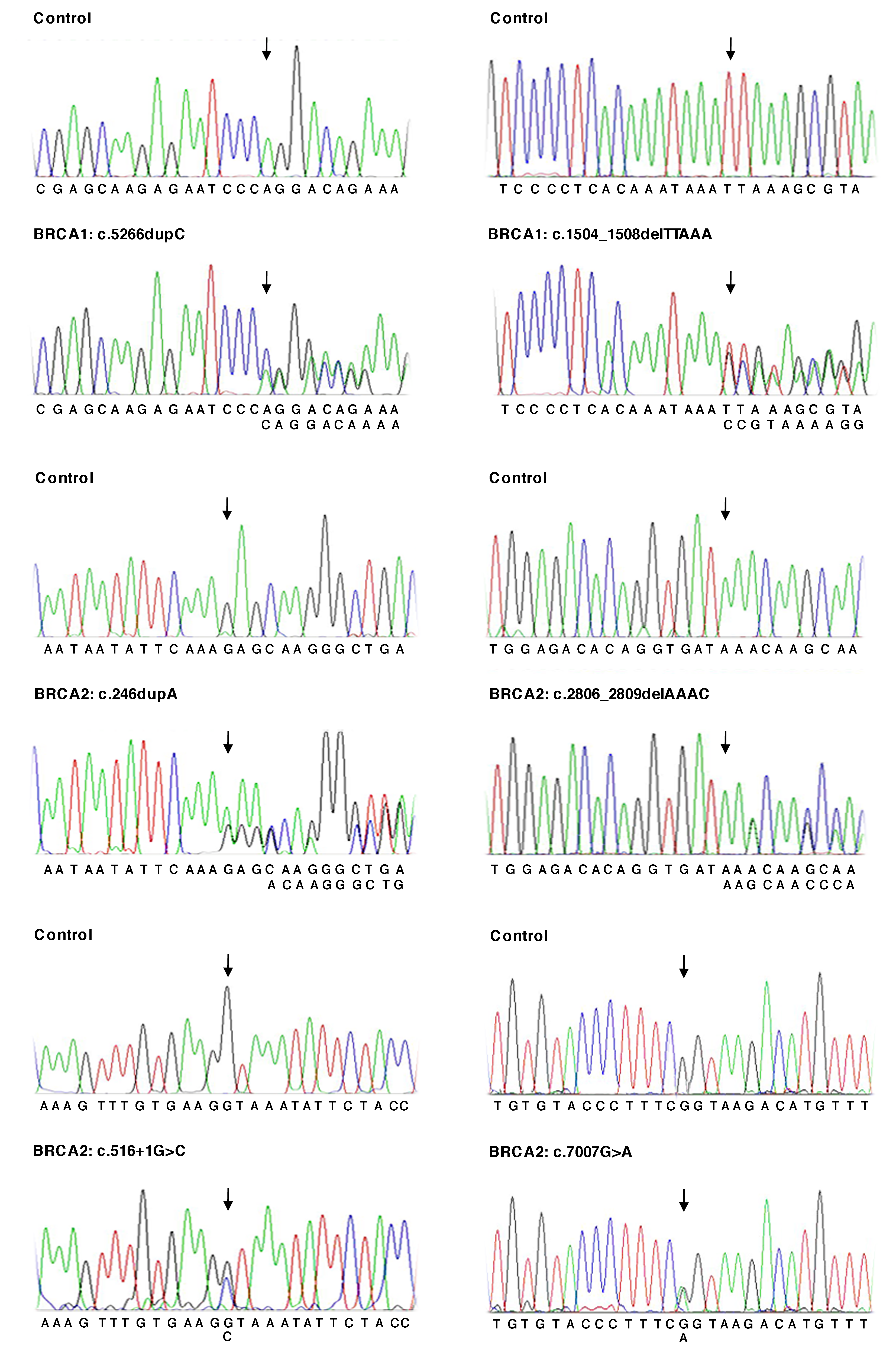

Supplement: Supplemental Information 5 — Electropherograms showing the BRCA1 and BRCA2 variants contained in the training set compared to a group of wild type samples. Arrows indicate the position of the mutated nucleotides. [file peerj-07-6661-s005.png]

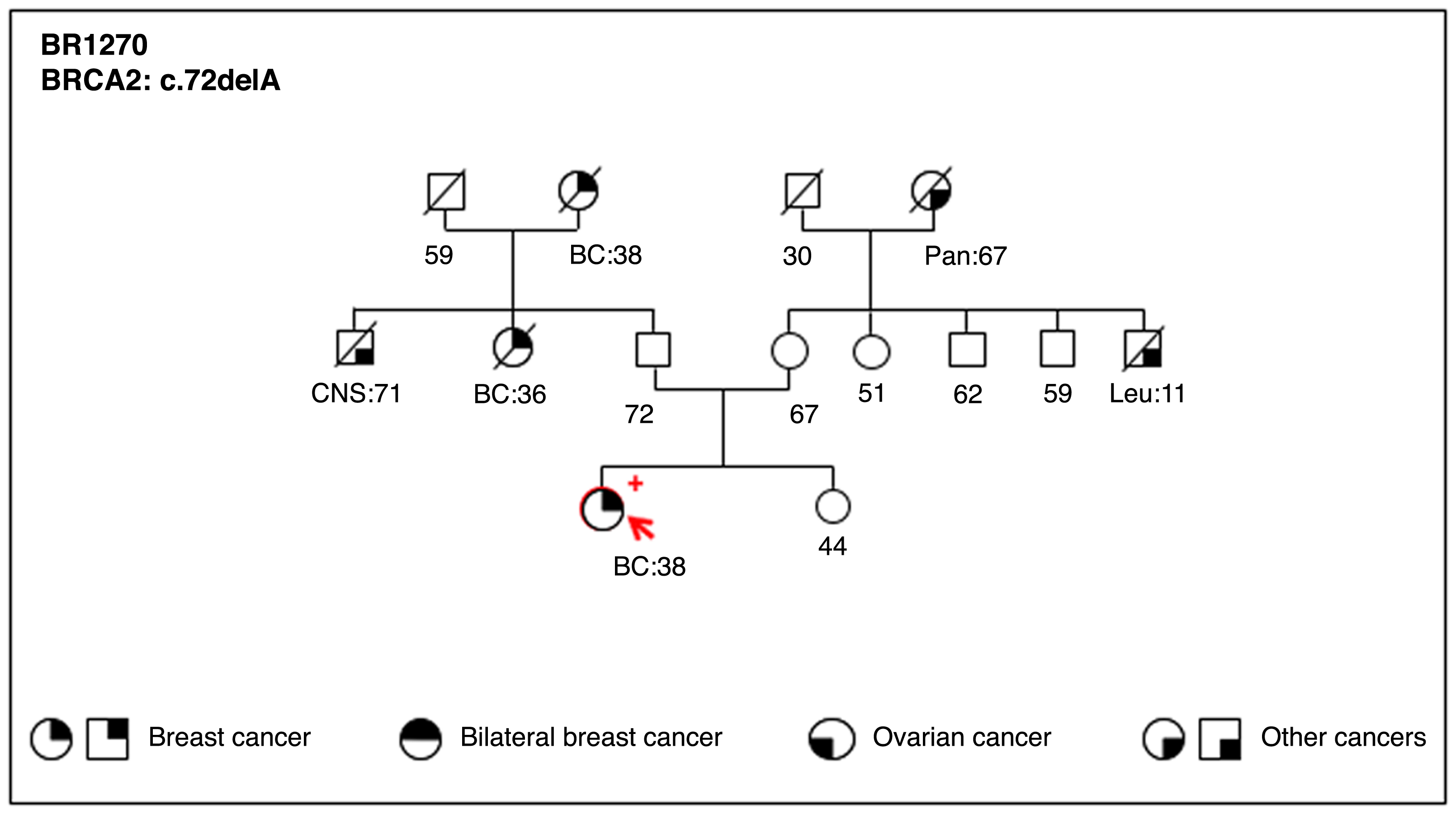

Supplement: Supplemental Information 6 — Proband, indicated with an arrow, is a carrier of the novel germline mutation at exon 3 of BRCA2 gene, c.72delA, identified by NGS. This disease causing variant is predicted to code for an early truncated protein (p.Gly25Aspfs). Cancer type and age at diagnosis are reported and described as: BC, breast cancer; Pan, pancreas; CNS, central nervous system cancer; Leu, leukemia. [file peerj-07-6661-s006.png]
